# CD4^+^ T Cell Hyporesponsiveness after Repeated Exposure to Schistosoma mansoni Larvae Is Dependent upon Interleukin-10

**DOI:** 10.1128/IAI.02831-14

**Published:** 2015-03-17

**Authors:** Catriona T. Prendergast, David E. Sanin, Peter C. Cook, Adrian P. Mountford

**Affiliations:** Centre for Immunology and Infection, Department of Biology, University of York, York, United Kingdom

## Abstract

The effect that multiple percutaneous exposures to Schistosoma larvae has on the development of early CD4^+^ lymphocyte reactivity is unclear, yet it is important in the context of humans living in areas where schistosomiasis is endemic. In a murine model of multiple infections, we show that exposure of mice to repeated doses (4×) of Schistosoma mansoni cercariae, compared to a single dose (1×), results in CD4^+^ T cell hyporesponsiveness within the skin-draining lymph nodes (sdLN), manifested as reduced CD4^+^ cell proliferation and cytokine production. FoxP3^+^ CD4^+^ regulatory T cells were present in similar numbers in the sdLN of 4× and 1× mice and thus are unlikely to have a role in effecting hyporesponsiveness. Moreover, anergy of the CD4^+^ cell population from 4× mice was slight, as proliferation was only partly circumvented through the *in vitro* addition of exogenous interleukin-2 (IL-2), and the *in vivo* blockade of the regulatory molecule PD1 had a minimal effect on restoring responsiveness. In contrast, IL-10 was observed to be critical in mediating hyporesponsiveness, as CD4^+^ cells from the sdLN of 4× mice deficient for IL-10 were readily able to proliferate, unlike those from 4× wild-type cohorts. CD4^+^ cells from the sdLN of 4× mice exhibited higher levels of apoptosis and cell death, but in the absence of IL-10, there was significantly less cell death. Combined, our data show that IL-10 is a key factor in the development of CD4^+^ T cell hyporesponsiveness after repeated parasite exposure involving CD4^+^ cell apoptosis.

## INTRODUCTION

Schistosomiasis is a disease caused by parasitic helminths of Schistosoma sp. and affects ∼230 million people worldwide (1, 2), with a further 779 million people at risk of infection (3, 4). In regions of endemicity, individuals are liable to be repeatedly exposed to free-swimming infective Schistosoma cercariae, resulting in multiple infections. Consequently, analyses of human immune responses to schistosomes are likely to be based upon individuals who have been exposed to multiple doses of excretory/secretory (E/S) material released by infectious larvae as well as other life cycle stages (e.g., eggs). Individuals with chronic schistosomiasis tend to develop a downregulated adaptive immune response (e.g., see references 5–7), which may be due to repeated exposure to infective larvae and/or long-term exposure to adult worms and eggs. In the former situation, infective cercariae release abundant E/S material originating from the glycocalyx and acetabular glands (8), which have immune-downregulatory activity (9–12). Indeed, whole-blood cultures from infected individuals from an area in northern Senegal where schistosomiasis is endemic secrete larger quantities of regulatory interleukin-10 (IL-10) in response to cercarial E/S material than do those from uninfected individuals (13). However, it is not known to what extent immune downregulation is caused by repeated exposure to infective cercariae and their E/S antigens.

In order to investigate the development of innate and acquired immune responses following repeated exposure to infective cercariae prior to the onset of egg deposition from adult worms, we developed a murine model of multiple schistosome infections (14). We reported that multiple exposures (4×) of the skin to infective schistosome cercariae resulted in CD4^+^ T cells in the skin-draining lymph nodes (sdLN) becoming hyporesponsive to antigen stimulation, in terms of their ability to proliferate and secrete cytokines, which developed before the presence of eggs in the hepatic portal system (14). The hyporesponsive state was systemic and led to a subsequent downmodulation of granulomatous immunopathology to eggs in the liver (14). Clearly, repeated exposure of the host to schistosome cercariae has an immunomodulatory effect, independent of egg deposition, but the mechanism(s) that underpins CD4^+^ T cell hyporesponsiveness induced by repeated exposure to schistosome larvae is not known.

CD4^+^ cell hyporesponsiveness caused by parasitic infections (15–17), particularly of Th2 lymphocytes due to chronic helminth infection, is well established (18–20). Typically, it manifests as an inability of antigen-specific cells to proliferate upon antigen restimulation and a failure to release specific cytokines (e.g., gamma interferon [IFN-γ] and IL-5). Various mechanisms of hyporesponsiveness have been proposed, including those intrinsic to the antigen-specific CD4^+^ lymphocyte population (e.g., anergy, exhaustion, or apoptosis) as well as extrinsic factors (e.g., inhibition by FoxP3^+^ CD4^+^ regulatory T [T_reg_] cells or regulatory IL-10). The lack of responsiveness by antigen-specific CD4^+^ lymphocytes has traditionally been referred to as anergy when the cells are rechallenged with antigen but in the absence of positive costimulation, e.g., via CD28 (21, 22). Exhaustion of CD8^+^ and CD4^+^ lymphocytes has been described following exposure to persistent/chronic infection with viruses (23) as well as several parasitic protozoa (17), especially where the host is exposed to a high antigenic load. These mechanisms are associated with various coinhibitory receptors, such as programmed cell death 1 (PD1) (24). Another aspect that could contribute to hyporesponsiveness is the induction of activation-induced cell death (AICD) or apoptosis in the T cell population, particularly through the engagement of Fas/FasL (25, 26). The importance of anergy, exhaustion, and/or AICD in the development of CD4^+^ cell hyporesponsiveness following repeated exposure to infective schistosome larvae is unknown, but others have suggested that CD11b^+^ macrophages acting as antigen-presenting cells (APCs) are modulated by prepatent schistosome worms (27). Finally, while extrinsic mechanisms of CD4^+^ cell hyporesponsiveness, such as CD4^+^ T_reg_ cells (28–30) or regulatory IL-10 (31–33), have been explored in the context of chronic schistosome infection (i.e., in the presence of eggs), they have not been investigated following repeated exposure to schistosome larvae prior to the production of eggs.

Here, we conducted a closer examination of the immune responses in the sdLN of mice exposed to 4× versus 1× doses of Schistosoma mansoni cercariae, in order to determine the relative contributions of the different mechanisms mentioned above that may result in CD4^+^ T cell hyporesponsiveness. We show that CD4^+^ T cells from 4× mice displayed a greater propensity to become anergic and were more likely to enter apoptosis, resulting in increased numbers of dead cells. We conclude that the early development of CD4^+^ T cell hyporesponsiveness in the host is dependent primarily upon the presence of regulatory IL-10 derived from CD4^+^ cells in the sdLN induced by repeated exposure to infectious schistosome larvae.

## MATERIALS AND METHODS

### Animals.

Wild-type (WT) C57BL/6 and IL-10-deficient (knockout [KO]) mice (34), transgenic mice expressing an A^b^-restricted ovalbumin peptide (pOVA)-reactive T cell receptor (TCR) on a RAG^−/−^ background (OT-II) (35), and IL-10 reporter knock-in (*tiger*) mice (36) were bred and housed at the University of York. Female mice aged between 6 and 10 weeks were used for all experiments, which were carried out in accordance with the United Kingdom Animals Scientific Procedures Act 1986 and with approval of the University of York Ethics Committee.

### Infection protocol and parasites.

The life cycle of a Puerto Rican strain of S. mansoni was maintained at the University of York by routine passage through outbred NMRI mice and Biomphalaria glabrata snails. Mice used for experiments were percutaneously exposed via each pinna to multiple doses (4×) of 150 S. mansoni cercariae at weekly intervals from day 0 to day 21, as previously described (14, 37). Age- and sex-matched cohorts were exposed in parallel to a single dose (1×) of 150 cercariae on day 21. By using this infection protocol, via the pinna, a 50% penetration rate is observed (37), amounting to 75 cercariae per pinna, or 150 cercariae per mouse, at each time point. In order to control for the infection dose, in some experiments, 1× mice were exposed to either a single low dose (150 cercariae) or a single high dose (600 cercariae). In all experiments, auricular lymph nodes draining the skin site of infection (sdLN) were harvested 4 days after infection or, in selected experiments for 1× mice, 25 days after infection [yielding the infection group 1× (d25)].

### *In vitro* culture of total sdLN cells.

A soluble schistosomula antigen preparation (SSAP) was prepared from *in vitro*-cultured, mechanically transformed larvae, as described previously (38), in order to stimulate sdLN cells for an antigen-specific recall response (37). Single-cell suspensions from the sdLN were cultured at 1 × 10^6^ cells/ml in RPMI 1640 medium containing 10% heat-inactivated fetal calf serum (FCS) (Biosera), 2 mM l-glutamine, 50 U/ml penicillin, 50 μg/ml streptomycin, and 50 μM 2-mercaptoethanol (all from Gibco) in the presence or absence of 50 μg/ml SSAP for 72 h at 37°C. Proliferation was measured either by [^3^H]thymidine incorporation (18.5 kBq per well) (39) or after labeling with 3 μM carboxyfluorescein diacetate succinimidyl ester (CFSE) (Invitrogen) (14). In the latter situation, cells were subsequently labeled with an anti-CD4 monoclonal antibody (MAb), and proliferation was determined by flow cytometry, judged according to the decrease in the fluorescence of CFSE.

### Cytokine analysis by ELISA.

Culture supernatants were collected from the *in vitro* sdLN cell cultures at 72 h for cytokine analysis, as described previously (40). The amounts of IL-4 were determined by using DuoSet enzyme-linked immunosorbent assay (ELISA) kits (R&D Systems). Amounts of IFN-γ were measured by using capture and detection antibodies (BD Pharmingen).

### *In vivo* cell proliferation.

*In vivo* cell proliferation was determined by bromodeoxyuridine (BrdU) incorporation. Mice received 1 mg BrdU (Sigma) intraperitoneally (i.p.) daily for the final 4 days before harvest of the sdLN. The sdLN cells were then processed to a single-cell suspension and blocked with anti-CD16/32 MAbs (eBioscience) in goat serum (Sigma) and later surface stained for anti-CD3 eF450 and anti-CD4 phycoerythrin (PE)-Cy7 (both from eBioscience) in phosphate-buffered saline (PBS) supplemented with 1% FCS. Subsequently, cells were washed in PBS–1% FCS and incubated in 1× Fixation/Permeabilization buffer (eBioscience) for at least 1 h at 4°C. Cells were then washed in PBS–1% FCS and incubated at 37°C in 100 μg DNase (Sigma) for 1 h. Afterwards, cells were washed in PBS–1% FCS and stained for 45 min at room temperature with anti-BrdU allophycocyanin or rat IgG1 allophycocyanin (eBioscience) in 1× permeabilization buffer, according to the manufacturer's protocol.

### Flow cytometry.

Cells were incubated with anti-CD16/32 MAbs (eBioscience) in goat serum (Sigma) to block the nonspecific uptake of antibodies and then subsequently labeled with the following MAbs conjugated to fluorescent labels: anti-CD4, anti-CD3, anti-CD8, anti-B220, anti-CD25, anti-CD103, anti-PD1, anti-PDL-1, anti-PDL-2 PE, anti-Fas, anti-FasL, anti-CD69, anti-CD44, anti-CD62L, anti-major histocompatibility complex class II (MHC-II) (IA-IE), anti-Nrp1, anti-CD11b (all from eBioscience), anti-CD49b (BioLegend), and anti-LAG3 (CD223) (BD Bioscience). Intracellular staining was performed according to the manufacturer's protocol for anti-FoxP3, anti-Helios, and anti-cytotoxic-T lymphocyte-associated antigen 4 (CTLA-4) (eBioscience). All flow cytometry data were acquired by using the Cyan ADP analyzer or the BD LSR Fortessa analyzer. Flow data were analyzed by using FlowJo software v7.6.5 (Tree Star, Inc.) or Summit v4.3 (DakoCytomation).

### Blockade of PD1.

In some experiments, the expression of PD1 was blocked through the use of an anti-PD1 antibody (RMP1-14) (BioXCell). Repeatedly infected mice (4× mice) received 500 μg i.p. in a 200-μl volume weekly, 1 day before each infection time point. The singly infected mice (1× mice) received one dose of 500 μg i.p. 1 day prior to infection. Control mice received 200 μl PBS i.p.

### Annexin V assay.

Cells were stained for the cell surface markers CD3 and CD4 as described above. Subsequently, the cells were washed in cold PBS supplemented with 1× cold annexin V binding buffer (eBioscience)and were then incubated for 15 min with anti-annexin V fluorescein isothiocyanate (FITC) at room temperature and finally washed in annexin V binding buffer. Cells were then resuspended in annexin V binding buffer for analysis, and propidium iodide (PI) (eBioscience) was added directly before acquiring data on the Cyan ADP analyzer.

### CD4^+^ cell sorting and *in vitr*o coculture with antigen-presenting cells.

Cells from the sdLN of 1× and 4× WT and IL-10-deficient mice were prepared as a single-cell suspension and labeled with antibodies against CD45, CD3, CD4, and B220, as described above. The cells were then sorted into CD45^+^ CD3^+^ CD4^+^ T cells and CD45^+^ CD3^−^ CD4^−^ B220^−^ APC populations by using a fluorescence-activated cell sorter (FACS) (MoFlo Astrios; Beckman Coulter). The CD3^+^ CD4^+^ T cells were labeled with 3 μM CFSE (Invitrogen), as described above, and then stimulated with 50 μg/ml SSAP for 72 h as cocultures of T cells (5 × 10^4^ cells) and APCs (1 × 10^4^ cells) in combinations from different groups of mice. Antigen-specific proliferation was measured by a decrease in the expression of CFSE after 72 h, as described above.

### Real-time quantitative PCR.

Cells from the sdLN were washed in cold PBS, resuspended in TRIzol (Invitrogen), and stored at −80°C. Total RNA was extracted, cDNA was generated by using Superscript II DNA polymerase (Invitrogen), and real-time quantitative PCR (qRT-PCR) was performed by using the StepOne Plus system (Applied Biosystems) with SYBR green (Applied Biosystems). The expression levels of genes were determined relative to the level of the housekeeping gene glyceraldehyde-3-phosphate dehydrogenase (GAPDH) by using the ΔΔ*C_T_* method. Primers used were GAPDH forward primer CCATGTTTGTGATGGGTGTG, GAPDH reverse primer CCTTCCACAATGCCAAAGTT, Fas forward primer CTACTGCGATTCTCCTGGCT, Fas reverse primer GGTTCCATGTTCACACGAGG, FasL forward primer CTCCGTGAGTTCACCAACCA, and FasL reverse primer TAAATGGGCCACACTCCTCG.

### Statistics.

Statistical analyses were performed by using Student's *t* test, the Mann-Whitney U test, one-way analysis of variance (ANOVA), or a Kruskal-Wallis test. GraphPad Prism v5 software was used to perform the statistical analyses (GraphPad Software, Inc.).

## RESULTS

### CD4^+^ T cell hyporesponsiveness in sdLN is due to repeated exposures to S. mansoni cercariae.

**FIG 1:**
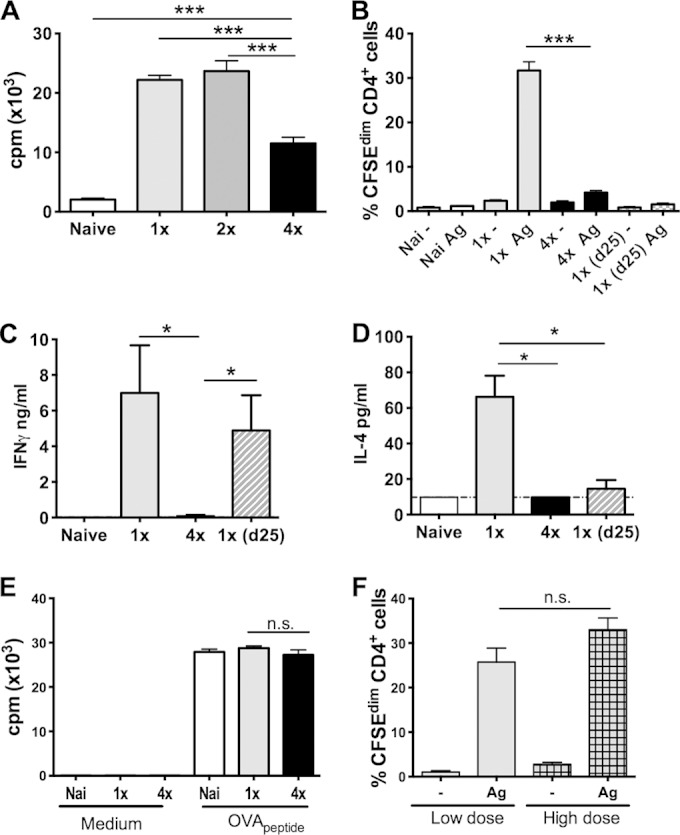
Lymphocyte hyporesponsiveness in sdLN is due to repeated exposure to S. mansoni cercariae. Mice were exposed to one (1×), two (2×), or four (4×) doses of 150 cercariae via percutaneous infection of the pinna at 7-day intervals, and sdLN were recovered 4 days after the final exposure. (A) Proliferation of sdLN cells from 1×, 2×, or 4× versus naive mice stimulated with SSAP *in vitro* prior to determination of proliferation, measured by the uptake of [^3^H]thymidine (cpm in the presence of SSAP minus cpm in medium only). (B) Proliferation of sdLN cells as a proportion of CFSE^dim^ CD4^+^ cells cultured *in vitro* in the presence or absence of SSAP. Groups shown are naive (Nai), 1×, 4×, and 1× (d25) mice. Ag, antigen. (C and D) Detection of IFN-γ (C) and IL-4 (D) secreted by *in vitro*-cultured sdLN cells in response to SSAP stimulation, as measured by cytokine-specific ELISAs. The horizontal dotted line represents the minimum level of detection. (E) Proliferation of sdLN cells from schistosome-infected transgenic OT-II mice stimulated with medium or ovalbumin peptide. (F) Proliferation of CD4^+^ cells from mice exposed to a single low dose (150 cercariae) or a high dose (600 cercariae), shown as a proportion of CFSE^dim^ CD4^+^ cells following restimulation *in vitro* in the presence or absence of SSAP. Statistical significance was determined by using the Mann-Whitney U test (*, *P* < 0.05; **, *P* < 0.01; ***, *P* < 0.001; n.s., not significant). Data are representative of results from 2 to 5 independent experiments (*n* = 4 to 6 mice).

The proliferation of *in vitro*-cultured total sdLN cells in response to soluble larval parasite antigen (SSAP) was increased in cells recovered from both 1× and 2× mice 4 days after the final infection (Fig. 1A). However, after four exposures (4×) to S. mansoni cercariae, total sdLN cells from 4× mice exhibited significantly lower levels of proliferation (*P* < 0.001) (Fig. 1A). Hyporesponsiveness was evident in the CD4^+^ T cell population, as CD4^+^ T cells from 4× mice divided to a much lesser extent than did CD4^+^ T cells from 1× mice (*P* < 0.001) (Fig. 1B), and was not simply confined to cell proliferation, as IFN-γ and IL-4 cytokine production from antigen-stimulated *in vitro*-cultured cells was also significantly reduced in 4× compared to 1× mice (*P* < 0.05) (Fig. 1C and D). Moreover, although CD4^+^ cells from 1× mice harvested on day 25 (cf. day 4) also exhibited low levels of proliferation (*P* > 0.05) (cf. 4× or naive cells) (Fig. 1B), this reflects the disappearance of antigen-specific cells in the absence of further antigen stimulation *in vivo* rather than the development of hyporesponsiveness, as 1× (d25) CD4^+^ cells released substantial amounts of IFN-γ in response to parasite antigen (*P* < 0.01) (Fig. 1C).

Hyporesponsiveness was specific to parasite antigen, since sdLN cells from uninfected OT-II mice (in which CD4^+^ T cells express a TCR specific to the OVA_323–339_ peptide) and those exposed to either 1× or 4× doses of schistosome cercariae all had similar highly elevated levels of *in vitro* proliferation in response to OVA peptide (Fig. 1E). Furthermore, hyporesponsiveness was not dependent on the number of parasites used at infection, since the proportion of proliferating CD4^+^ cells from mice exposed to a single low dose of cercariae was not significantly different from that from mice exposed to a single high dose equivalent to the cumulative dose experienced by 4× mice (*P* > 0.05) (Fig. 1F). Cells from mice exposed to either a low or high dose of cercariae also secreted large quantities of IFN-γ and IL-4 (see Fig. S1A and S1B in the supplemental material), showing that the infection dose is not a factor in the induction of lymphocyte hyporesponsiveness.

### Infection with S. mansoni increases lymphocyte cellularity of sdLN.

**FIG 2:**
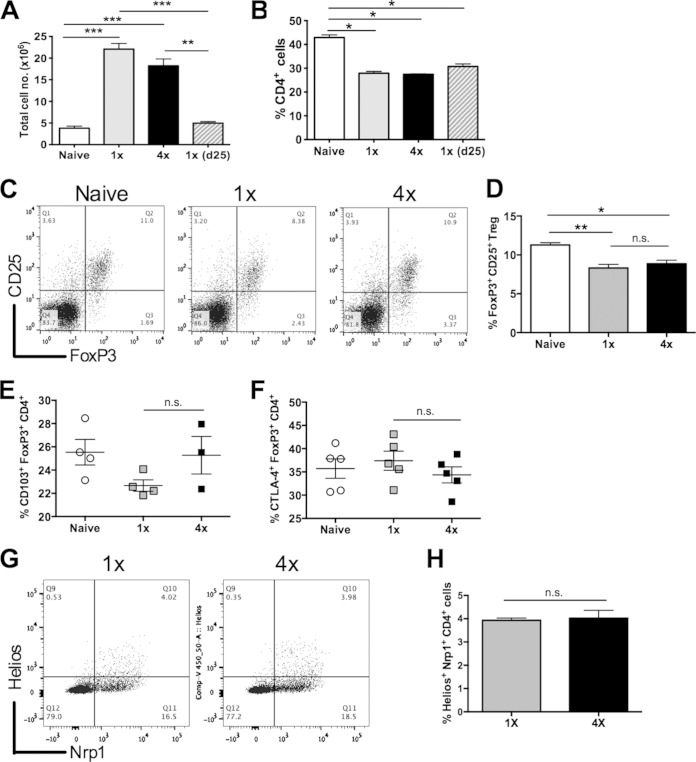
The proportion of FoxP3^+^ regulatory T cells does not change between 1× and 4× mice. (A and B) Total cell numbers from the sdLN (A) and proportion of sdLN CD4^+^ cells (B), as determined by flow cytometry. (C) Representative flow cytometry dot plots showing the gating strategy for FoxP3^+^ and CD25^+^ CD4^+^ sdLN cells from naive, 1×, and 4× mice obtained 4 days after infection, previously gated on CD4^+^ T cells. (D) FoxP3^+^ CD4^+^ T_reg_ cells as a proportion of total CD4^+^ cells. The means and standard errors of the means for groups of naive, 1×, and 4× mice (*n* = 5) are shown. (E and F) Expression of CD103^+^ (E) and CTLA-4^+^ FoxP3^+^ CD4^+^ (F) cells. Symbols are percent expression values for individual mice (*n* = 4 or 5); horizontal bars represent the means ± standard errors of the means. (G) Representative flow cytometry dot plots for CD4^+^ cells labeled with anti-Nrp1 and anti-Helios antibodies. Values in the upper right quadrant represent the proportions of thymic Nrp1^+^ Helios^+^ CD4^+^ cells in the sdLN from 1× and 4× mice. (H) Nrp1^+^ Helios^+^ CD4^+^ T_reg_ cells as a proportion of total CD4^+^ cells. Means and standard errors of the means for groups of 1× and 4× mice (*n* = 5) are shown. Statistical significance was determined by using the Mann-Whitney U test (n.s., *P* > 0.05; *, *P* < 0.05; **, *P* < 0.01; ***, *P* < 0.001).

The total numbers of cells recovered from the sdLN of both 1× and 4× mice 4 days after the last infection were significantly increased compared to those of the naive group (Fig. 2A). Conversely, the number of sdLN cells from 1× mice obtained 25 days after infection was low and not significantly different from that of the naive group (*P* > 0.05) (Fig. 2A). The proportions of cells that were CD4^+^ in the sdLN of 1× and 4× mice were much lower than those in naive mice (*P* < 0.05) (Fig. 2B) but not significantly different between the two groups of infected mice. The proportions of CD8^+^ and B220^+^ cells also did not differ between 1× and 4× mice (*P* > 0.05) (see Fig. S1C and S1D in the supplemental material), indicating that exposure to repeated doses versus a single dose of cercariae does not have a major effect upon the different types of lymphocytes found in the sdLN. In both groups of infected mice, B cells probably account for most of the increased sdLN cellularity compared to naive animals.

### The proportion of FoxP3^+^ regulatory T cells does not change between 1× and 4× mice.

An increase in the proportion of FoxP3^+^ regulatory T (T_reg_) cells could account for the hyporesponsive state observed in CD4^+^ cells from 4× compared to 1× mice. However, the proportions of FoxP3^+^ CD25^+^ T_reg_ cells in sdLN were similar in 1× and 4× mice (*P* > 0.05) (Fig. 2C and D). In addition, the expression levels of CD103 (integrin αEβ7), which provides an indication of the functional status of FoxP3^+^ T_reg_ cells (41, 42), were equivalent in FoxP3^+^ cells from naive, 1×, and 4× mice (Fig. 2E), as were the expression levels of intracellular CTLA-4, thought to be crucial for the function and suppressive ability of FoxP3^+^ T_reg_ cells (43) (Fig. 2F). Thymic FoxP3^+^ T_reg_ cells can be differentiated from peripheral FoxP3^+^ T_reg_ cells through their expression of neuropilin 1 (Nrp1) and the transcription factor Helios (44, 45). Nevertheless, the proportions of Helios^+^ Nrp1^+^ CD4^+^ thymic T_reg_ cells also did not differ between the sdLN of 1× and 4× mice (*P* > 0.05) (Fig. 2G and H), and therefore, together, our data do not support a role for increased numbers of FoxP3^+^ T_reg_ cells in the sdLN of 4× mice as the cause of CD4^+^ lymphocyte hyporesponsiveness.

### A proportion of CD4^+^ cells from 4× mice are anergic.

**FIG 3:**
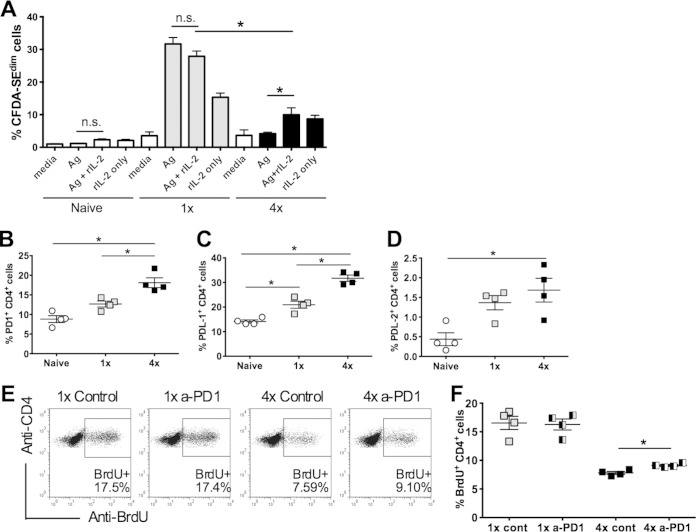
Anergy in CD4^+^ T cells from 4× mice. (A) Cells from sdLN of naive, 1×, and 4× mice were cultured *in vitro* and stimulated with medium, SSAP, exogenous IL-2 only, or SSAP and IL-2 combined. The proliferation of CD4^+^ cells is shown as a proportion of CFSE^dim^ CD4^+^ cells. rIL-2, recombinant IL-2. (B to D) Expression of PD1 (B), PDL-1 (C), and PDL-2 (C) on CD4^+^ cells in sdLN from naive, 1×, and 4× mice, expressed as a proportion of total CD4^+^ cells. Symbols are percent expression values for individual mice; horizontal bars represent means ± standard errors of the means (*n* = 4 mice). (E) Representative flow cytometry dot plots showing expression of BrdU versus CD4, previously gated on CD4^+^ T cells. The gated population denotes those cells that are both CD4^+^ and BrdU^+^. Cells from the sdLN were obtained from 1× and 4× mice given either regular injections of anti-PD1 antibody or control injections. (F) Graphical representation of data acquired (as described above for panel E) from groups of 4 individual mice, shown as the percentage of CD4^+^ BrdU^+^ cells. Symbols are percent expression values for individual mice; horizontal bars represent means ± standard errors of the means (*n* = 4 mice). Statistical significance was determined by using the Mann-Whitney U test (n.s., not significant; *, *P* < 0.05). Data are representative of results from 1 to 3 independent experiments.

In order to investigate whether anergy was a cause of hyporesponsiveness, sdLN cells from infected mice were cultured *in vitro* in the presence of parasite antigen, with or without the addition of exogenous recombinant IL-2. While CD4^+^ T cells from 1× mice proliferated strongly in response to SSAP, the level of proliferation was not further enhanced by the addition of IL-2 (Fig. 3A). On the other hand, CD4^+^ T cells from 4× mice, which hardly proliferated, were partly rescued from their hyporesponsive state following the addition of exogenous IL-2 *in vitro* compared to the antigen alone (*P* < 0.05) (Fig. 3A), although the addition of IL-2 did not restore proliferation to the levels recorded for antigen-stimulated cells from 1× mice (*P* < 0.001) (Fig. 3A).

Anergic cells are known to exhibit increased expression levels of programmed cell death marker 1 (PD1) and its ligands (PDL-1 and PDL-2) on a number of different cell types (46, 47). Indeed, the expression level of PD1 was increased on CD4^+^ cells from 4× compared to 1× mice (*P* < 0.05) (Fig. 3B), as was the expression level of PDL-1 (*P* < 0.05) (Fig. 3C). However, although the PDL-2 expression level was increased on CD4^+^ cells from 4× compared to naive mice (*P* < 0.05), it was not increased above the expression level in 1× mice (*P* > 0.05) (Fig. 3D). Expression levels of PD1 and PDL-1 on CD11b^+^ myeloid cells were not significantly increased in 4× compared to 1× mice (*P* > 0.05) (see Fig. S2A and S2B in the supplemental material), although the PDL-2 level was significantly higher on 4× than on 1× sdLN CD11b^+^ cells (*P* < 0.05) (see Fig. S2C in the supplemental material).

In order to determine if PD1 was required *in vivo* for the development of hyporesponsiveness, mice were administered anti-PD1 MAb throughout the infection process, and the proliferation of CD4^+^ T cells was determined *in vivo* by the uptake of BrdU (Fig. 3E). There was no difference in the numbers or proportions of CD4^+^ sdLN cells in mice after the PD1 blockade (data not shown). However, the proliferation of CD4^+^ cells of 4× mice following the blockade of PD1 signaling was marginally increased compared to that of 4× control mice (*P* < 0.05) (Fig. 3F). Conversely, the PD1 blockade had no effect on the proliferative capacity of CD4^+^ cells from 1× mice (Fig. 3F).

### CD4^+^ T cells from 4× mice exhibit increased levels of apoptosis.

**FIG 4:**
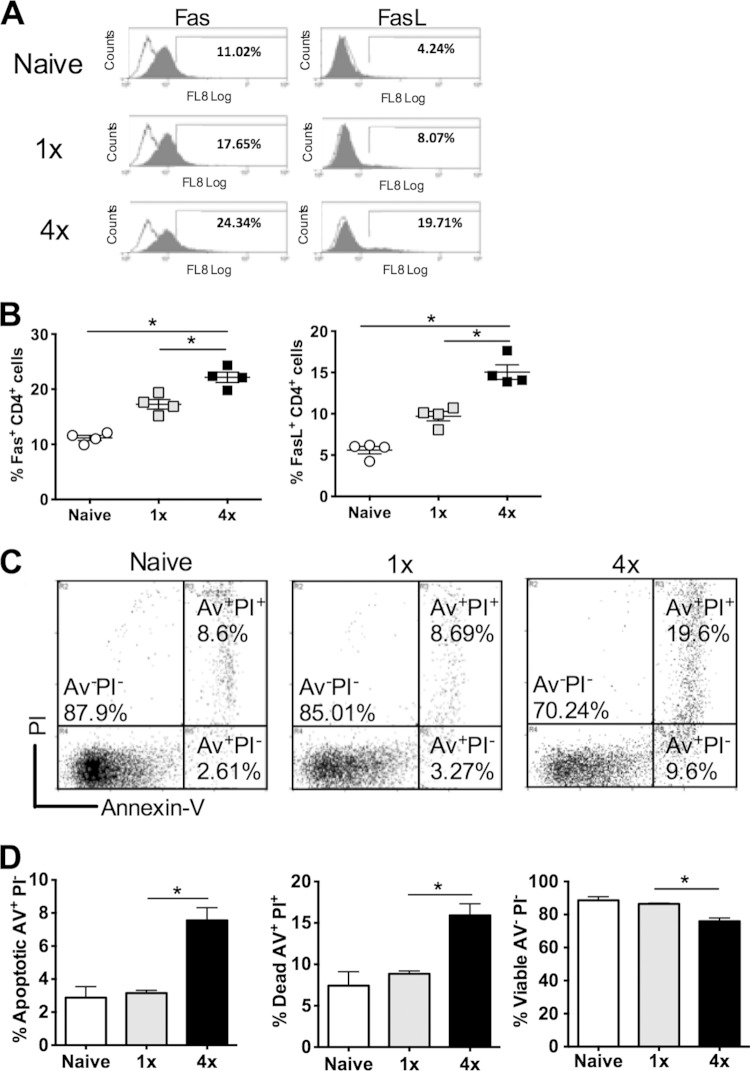
CD4^+^ T cells from 4× mice exhibit increased levels of apoptosis. (A) Representative flow cytograms showing the expression of Fas and FasL on CD4^+^ cells from sdLN. Solid gray histograms show positive labeling with specific antibodies, while open histograms represent labeling with the isotype control antibody. (B) Expression of Fas and FasL on CD4^+^ cells in sdLN, expressed as a proportion of total CD4^+^ cells. Symbols are percent expression values for individual mice; horizontal bars represent means ± standard errors of the means (*n* = 4 mice). (C) Representative flow cytometry dot plots showing the expression of annexin V (Av) versus PI on CD4^+^ T cells, where annexin V^+^ PI^−^ cells are apoptotic, annexin V^+^ PI^+^ cells are dead, and annexin V^−^ PI^−^ cells are live, from the sdLN of naive, 1×, or 4× mice. (D) Data from groups of mice are presented as bars representing the means and standard errors of the means for separate groups of mice (*n* = 4). Statistical significance was determined by using the Mann-Whitney U test (*, *P* < 0.05). Data are representative of results from 3 independent experiments.

Expression levels of Fas (CD95) and FasL (CD178), as markers of future cell death (48), were significantly increased on CD4^+^ T cells from the sdLN of 4× compared to 1× and naive mice (*P* < 0.05) (Fig. 4A and B), indicating that the engagement of Fas with FasL could facilitate elevated levels of AICD in the sdLN of 4× mice in response to repeated schistosome infection. Analysis of CD4^+^ cells according to positive staining for propidium iodide (PI) and annexin V (Fig. 4C) revealed that the proportion of annexin V-positive (annexin V^+^) PI-negative (PI^−^) apoptotic cells was highest in 4× mice, as was the proportion of annexin V^+^ PI^+^ dead cells (both *P* < 0.05) (Fig. 4D). This was reflected by a corresponding decrease in the proportion of viable annexin V^−^ PI^−^ CD4^+^ lymphocytes in 4× mice (*P* < 0.05) (Fig. 4D). Therefore, repeated infection with schistosome larvae leads to a significant increase in the proportion of apoptotic and dead CD4^+^ cells, which would affect the overall number of cells that can divide and secrete cytokines.

### Hyporesponsiveness in the CD4^+^ lymphocyte population is dependent upon the presence of IL-10.

**FIG 5:**
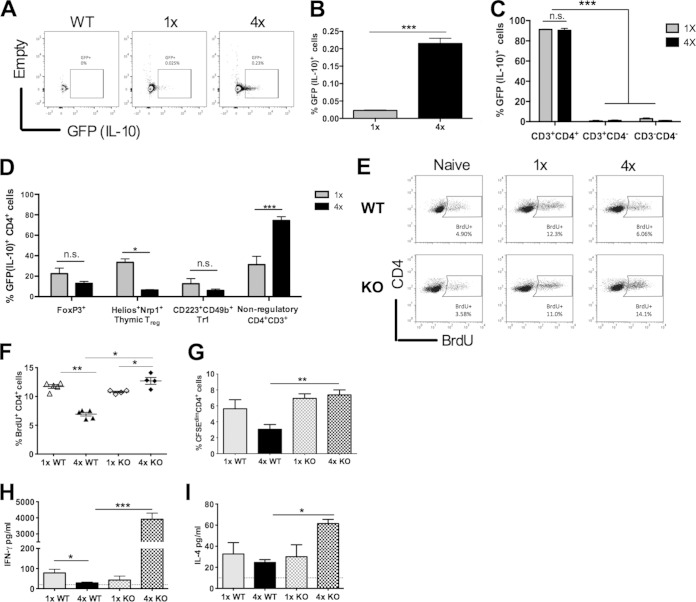
CD4^+^ cell hyporesponsiveness in sdLN is dependent upon IL-10. (A) Expression of IL-10 by sdLN cells from 1× and 4× IL-10 reporter knock-in (*tiger*) mice compared to 1× WT mice, shown as representative flow cytometry dot plots of all cells gated on GFP^+^ cells. (B) Bar chart showing the percentage of GFP/IL-10^+^ cells as a proportion of all sdLN cells. (C) Bar chart showing GFP/IL-10^+^ cells from 1× and 4× mice plotted according to their expression of CD3 and CD4. Values given are percentages of CD3^+^ CD4^+^, CD3^+^ CD4^−^, and CD3^−^ CD4^−^ cells. (D) Proportion of IL-10/GFP^+^ CD4^+^ cells, characterized as also being FoxP3^+^ (thymic and peripheral T_reg_ cells), Nrp1^+^ Helios^+^ (thymic T_reg_ cells), LAG3^+^ CD49b^+^ (Tr1 cells), or FoxP3^−^ Helios^−^ (nonregulatory cells). Bars represent means and standard errors of the means for groups of 1× and 4× mice (*n* = 5). (E) Number of cells that were dividing (i.e., BrdU^+^), as shown by representative flow cytometry dot plots for each group of mice. The gating strategy shows cells that were BrdU^+^. (F) Expression of BrdU on CD4^+^ cells in sdLN from 1× WT, 4× WT, 1× IL-10 KO, and 4× IL-10 KO mice as a proportion of total CD4^+^ cells. Symbols indicate the percent positive cells for individual mice; horizontal bars represent means ± standard errors of the means (*n* = 4 mice). (G) Proliferation of CD4^+^ cells shown as a proportion of CFSE^dim^ CD4^+^ cells for groups of 1× WT, 4× WT, 1× IL-10 KO, and 4× IL-10 KO mice. Cells were cultured *in vitro* in the presence or absence of SSAP. Bars show the means and standard errors of the means (*n* = 4). (H and I) Production of IFN-γ (H) and IL-4 (I) determined by cytokine-specific ELISAs. The dotted lines represent the minimum level of detection. For all experiments, statistical significance was determined by using the Mann-Whitney U test (n.s., *P* > 0.05; *, *P* < 0.05; **, *P* < 0.01; ***, *P* < 0.001). Data are representative of results from 2 to 4 independent experiments.

IL-10 is considered to be a master regulator of the immune response (reviewed in reference 49; 50, 51); therefore, we sought to determine whether it was an overriding factor in the development of CD4^+^ hyporesponsiveness. Using IL-10 reporter mice (36), the overall proportion of IL-10/green fluorescent protein-positive (GFP^+^) cells obtained directly *ex vivo* from the sdLN of infected mice in the absence of stimulation with parasite antigen, or phorbol myristate acetate (PMA), was low (Fig. 5A), although the proportion of IL-10/GFP^+^ cells in 4× mice was 10-fold higher than that in 1× mice (*P* < 0.001) (Fig. 5B). The majority (∼95%) of IL-10/GFP^+^ cells from the sdLN were CD3^+^ CD4^+^ T helper cells in both 4× and 1× mice, with only a small percentage being CD3^+^ CD4^−^ (probably CD8^+^ cells) or CD3^−^ CD4^−^ (which could be B cells or myeloid cells) (Fig. 5C). However, there was no difference in the proportion of IL-10-producing FoxP3^+^ CD4^+^ T_reg_ cells or in the proportion of IL-10-producing Tr1 cells (LAG3^+^ CD49b^+^) in 4× compared to 1× mice (*P* > 0.05) (Fig. 5D), although there was a reduction in the proportion of IL-10/GFP^+^ Nrp1^+^ Helios^+^ thymic T_reg_ cells (*P* < 0.05). Significantly, there were more IL-10/GFP^+^ CD4^+^ T cells of a nonregulatory phenotype (i.e., neither FoxP3^+^ T_reg_ nor Tr1 cells) in 4× than in 1× mice (*P* < 0.001) (Fig. 5D).

The increased presence of IL-10/GFP^+^ cells in 4× mice led us to determine the contribution of IL-10 in causing immune hyporesponsiveness in the sdLN of mice genetically deficient for IL-10. Measurement of BrdU *in vivo* uptake demonstrated that the proportions of proliferating CD4^+^ cells were similar in 1× WT and 1× IL-10 KO mice (Fig. 5E and F). However, while the proportion of dividing cells *in vivo* was, as expected, significantly reduced in 4× WT mice compared to 1× WT mice (*P* < 0.01), the proportion of dividing CD4^+^ cells in 4× KO mice was significantly higher than that in 4× WT mice and also higher than that in 1× KO mice (both *P* < 0.05) (Fig. 5F). The absence of hyporesponsiveness in 4× KO mice was further demonstrated *in vitro*, as CD4^+^ T cells from 4× KO mice proliferated in response to antigen restimulation to a much greater extent than did those from 4× WT mice (*P* < 0.01) (Fig. 5G). In addition, the amounts of IFN-γ secreted by sdLN cells from 4× KO mice were ∼100-fold larger than the amounts secreted by cells from 4× WT mice (*P* < 0.001) (Fig. 5H); the level of production of IL-4 was also higher in the sdLN of 4× KO mice (*P* < 0.05) (Fig. 5I). Consequently, in the absence of IL-10, CD4^+^ cells proliferate more vigorously than do cells from WT mice and have an enhanced ability to secrete both Th1 and Th2 cytokines, showing that IL-10 is essential for the development of the observed hyporesponsiveness. Moreover, the proportions of peripheral FoxP3^+^ T_reg_, Nrp1^+^ Helios^+^ thymic T_reg_, or LAG3^+^ CD49b^+^ Tr1 cells in the absence of IL-10 (i.e., between 4× WT and 4× KO mice) were not significantly different (data not shown), supporting our above-described observations which showed a lack of evidence for conventional CD4^+^ T_reg_ cells as a cause of hyporesponsiveness in our infection model.

As IL-10 conventionally downregulates the activation status of cells with antigen-presenting function (50), putative APCs from IL-10 KO sdLN could be functionally more efficient at driving CD4^+^ cell proliferation. However, while the proliferation of CD4^+^ cells from 1× WT sdLN was supported by putative endogenous APC populations recovered from the sdLN of 1× WT, 4× WT, and 4× KO mice, CD4^+^ cells from the sdLN of 4× WT mice exhibited lower levels of proliferation upon coculture with APCs from 1× WT, 4× WT, and 4× KO mice (*P* < 0.001) (see Fig. S3 in the supplemental material).

### Absence of IL-10 in 4× mice leads to enhanced CD4^+^ cell activation and a decline in apoptosis.

**FIG 6:**
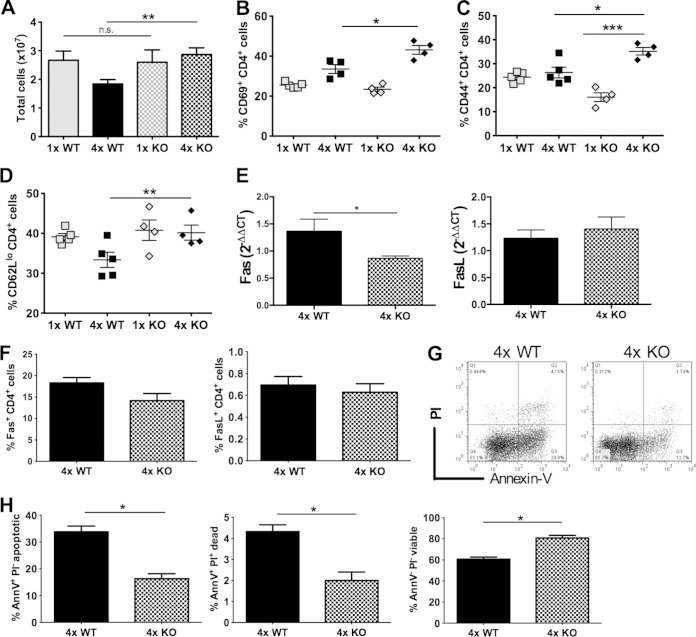
CD4^+^ cells in sdLN of 4× IL-10 KO mice have an enhanced activation status and are less prone to apoptosis. (A) Total number of viable sdLN cells in 1× WT, 4× WT, 1× KO, and 4× KO mice. Bars show means and standard errors of the means (*n* = 4 to 5). (B to D) Expression of CD69 (B), CD44 (C), and CD62^lo^ (D) on CD4^+^ cells in sdLN from 1× and 4× WT or KO mice, expressed as a proportion of total CD4^+^ cells from each group of mice. Symbols are percent expression values for individual mice; horizontal bars show the means ± standard errors of the means (*n* = 4 mice). (E and F) Transcript analysis by quantitative PCR for Fas (left) and FasL (right) in mRNA from the sdLN (E) and expression of Fas (left) and FasL (right) on CD4^+^ cells from the sdLN of 4× WT and 4× KO mice, expressed as a proportion of total CD4^+^ cells from each group of mice (F). (G) Representative flow dot plots for the sdLN of 4× WT and 4× KO mice showing the expression of annexin V versus PI, where annexin V^+^ PI^−^ cells are apoptotic, annexin V^+^ PI^+^ are dead, and annexin V^−^ PI^−^ cells are live. (H) Total proportions of annexin V^+^ PI^−^, annexin V^+^ PI^+^, and annexin V^−^ PI^−^ cells from 4× WT and 4× KO mice. Data from groups of mice are presented as bar charts, with bars representing the means and standard errors of the means (*n* = 4 mice). Statistical significance was determined by using the Mann-Whitney U test (n.s., not significant; *, *P* < 0.05; **, *P* < 0.01; ***, *P* < 0.001). Data are representative of results from 3 independent experiments.

In the sdLN, there was a significantly increased number of cells in 4× KO compared to 4× WT mice (*P* < 0.01) (Fig. 6A). The expression levels of the activation markers CD69 and CD44 on CD4^+^ T cells in the sdLN were also significantly higher in 4× KO mice (both *P* < 0.05) (Fig. 6B and C), while the proportion of CD62L^lo^ CD4^+^ T cells was significantly increased (*P* < 0.01) (Fig. 6D). Conversely, there were no significant differences in the expression levels of PD1 and PDL-2 between 4× WT and KO mice, although the level of PDL-1 was marginally higher in KO mice (data not shown). Analysis of mRNA transcripts showed that while there was a significant decrease in Fas expression the sdLN obtained from 4× KO mice (*P* < 0.05) (Fig. 6E), indicating that there was less apoptosis in the absence of IL-10, there was no difference in the expression of FasL (*P* > 0.05) (Fig. 6E). Moreover, although there were no significant differences in the expression of these markers by flow cytometry for 4× KO compared to 4× WT mice (*P* > 0.05) (Fig. 6F), there was a trend toward decreased expression in KO mice.

CD4^+^ T cells in the sdLN of 4× WT compared to KO mice exhibited high levels of apoptosis and death (both *P* < 0.05) (Fig. 6G and H), which could account for the hyporesponsiveness observed for the WT CD4^+^ cell populations. Conversely, there was a significantly higher proportion of live/viable CD4^+^ T cells in the sdLN of 4× KO mice (*P* < 0.05) (Fig. 6H). Therefore, the presence of IL-10 has a bearing on the levels of apoptosis and cell death, which may therefore contribute to CD4^+^ lymphocyte hyporesponsiveness.

## DISCUSSION

We show that after a single (1×) exposure of mice to infective S. mansoni cercariae, CD4^+^ T cells from the sdLN proliferate vigorously *in vivo*, or *in vitro* upon parasite antigen restimulation, and that these cells secrete substantial amounts of IFN-γ and IL-4. However, after repeated infections (i.e., 4×), CD4^+^ cells were not able to proliferate or secrete cytokines in response to antigen restimulation. It is possible that CD4^+^ hyporesponsiveness is caused by exhaustion due to the presence of high levels of antigen (23) following four exposures to infective cercariae. However, this seems unlikely, since exposure of mice to one high-dose infection (600 cercariae), which is equivalent to the cumulative dose after four infections (i.e., 4× with 150 cercariae), resulted in a proliferative and cytokine response of a magnitude similar to that following a single dose of 150 cercariae. The hyporesponsive state was parasite antigen specific, as repeated exposure to infective parasites (i.e., 4× doses) did not affect the capacity of sdLN cells from OT-II mice to proliferate, but appeared to differ from data from previous studies of mice exposed to high antigenic loads due to prepatent schistosome worm infections, which caused hyporesponsiveness to schistosome as well as nonschistosome antigens (27).

An alternative explanation for the development of hyporesponsiveness is CD4^+^ cell intrinsic anergy (21, 22), as demonstrated in our model by the partial restoration of antigen-specific proliferation of *in vitro*-cultured 4× sdLN cells in the presence of exogenous recombinant IL-2. However, although the proliferation of 4× cells in the presence of IL-2 was significantly increased compared to that in 4× cells stimulated with antigen only, it was still well below the level of proliferation recorded for 1× sdLN cells (with or without additional IL-2). In addition, while the expression levels of the anergy-related factors PD1 and PDL-1 (24, 47) on CD4^+^ cells were higher in 4× than in 1× WT mice, there was no significant change in the expression level of PD1 in the absence of IL-10 despite the restoration of CD4 cell responsiveness. Furthermore, the administration of anti-PD1 antibody *in vivo* to 4× WT mice led to only a small increase in the ability of CD4^+^ T cells to proliferate. This contrasts with data from previous studies of parasitic helminth infections, where a blockade of PD1 during infection with Litomosoides sigmodontis led to a major restoration of Th2 responsiveness mediated by PDL-2 (52), or exposure to adult schistosome worms (prior to the Th2-polarized chronic phase of infection), where T cell anergy was induced via PDL-1-mediated regulation (53). Nevertheless, taken together, our data indicate that although anergy involving PD1 may be operational in our multiple-infection model, it does not appear to have a major role, and it is possible that other inhibitory factors associated with hyporesponsiveness may be involved. For example, LAG3 (54) and its expression along with CD49b have been reported in helminth-infected mice (55), and GRAIL (gene related to anergy in lymphocytes) has been linked to a decline in Th2 cell responsiveness due to repeated stimulation with schistosome egg antigens (56). Microarray analysis of CD4 T cells from 4× versus 1× mice might reveal anergy-associated genes in our model of repeated exposure to schistosome larvae.

While the number of CD25^+^ FoxP3^+^ T_reg_ cells rapidly increased in other models of helminth infection (41, 57, 58), there was no significant increase in the number of FoxP3^+^ T_reg_ cells as a proportion of CD4^+^ cells in 4× compared to 1× mice, nor were there differences in the expression levels of CD103 and CTLA-4 markers on FoxP3^+^ T_reg_ cells or in the abundances of thymic Helios^+^ Nrp1^+^ CD4^+^ cells discrete from peripheral T_reg_ cells. Moreover, as there were no differences in the frequencies of conventional T_reg_ populations in the absence of IL-10, and since the transfer of FoxP3^+^ CD4^+^ cells from 4× into 1× mice did not confer hyporesponsiveness to recipient mice (data not shown), we conclude that our data do not support a role for FoxP3^+^ T_reg_ cells at this early period in the sdLN. This finding agrees with data from a previous study of prepatent 4-week schistosome infections, where CD4^+^ cell hyporesponsiveness was also independent of T_reg_ cells (27). This is perhaps not unexpected, as it is normally thought that T_reg_ cells have a functional effect on the immune response to parasitic helminths following long-term chronic infection requiring the presence of persistent antigen (18), whereas in our model of repeat infection, hyporesponsiveness was evident within the first weeks after infection.

The major factor demonstrated to be critical for the development of hyporesponsiveness in our model is the cytokine IL-10. It is an important regulator of the adaptive immune response (50, 59) and has been described as a “master” of immune regulation in the context of infectious disease (49). IL-10 could be derived from lymphoid Th1 or Th2 cells as well as cells of myeloid origin, such as macrophages, dendritic cells, or mast cells (30–32, 50, 51, 55). Although we have previously shown that the level of secretion of IL-10 following 72 h of *in vitro* culture of sdLN cells in response to parasite antigen was markedly lower for 4× than for 1× cells (14), we demonstrate here that the majority of IL-10 detected within sdLN cells recovered directly *ex vivo* from 1× and 4× mice, without additional stimulation with antigen or PMA, is derived from CD4^+^ cells. However, this rare (∼0.25% of total sdLN cells from 4× mice) population of T cells does not proliferate or secrete abundant cytokines in response to antigen restimulation *in vitro*. While FoxP3^−^ Tr1 cells have been identified as the main source of CD4^+^ cell-derived IL-10 following chronic infection with other helminths (55, 60), they did not contribute greatly to the numbers of IL-10/GFP^+^ cells detected *ex vivo* in the present study. Therefore, we conclude that since the abundance of IL-10/GFP^+^ CD4^+^ cells in the sdLN of 4× mice was substantially increased compared to that in 1× cohort mice, the majority of IL-10 is derived from nonregulatory CD4^+^ cells (i.e., non-T_reg_ cells), which could be Th2 effector cells, but appears to be the primary cause of early-stage CD4^+^ cell hyporesponsiveness in the sdLN.

When hyporesponsive CD4^+^ cells from 4× WT mice were cocultured with endogenous APCs from the sdLN of responsive 4× KO mice, their ability to proliferate was not restored, suggesting that the hyporesponsive state is intrinsic to CD4^+^ T cell populations and not dependent upon the presence of extrinsic APCs. We previously showed that dermal cells with antigen-presenting function from the skin of mice exposed to multiple doses of infectious cercariae, a site which is rich in IL-10, had an impaired ability to support the proliferation of sdLN CD4^+^ cells from 1× mice (14). While this may seemingly be at odds with our current findings, we do not know whether the different types of potential APCs in the skin migrate to a site of immune priming in the sdLN in the same, or similar, proportions as those observed in the skin. Myeloid APCs in the skin of multiply infected mice had an M2-like phenotype (14), but we have found no evidence for alternatively activated macrophages in the sdLN after multiple infections (data not shown). Nevertheless, in the absence of IL-10, CD4^+^ sdLN cells from 4× KO mice have a more activated phenotype, illustrated by increased expressions of CD69 and CD44, leading to their enhanced ability to proliferate *in vivo* and *in vitro* and secrete IFN-γ in response to antigen-specific stimulation *in vitro*. In this respect, it is notable that the number of cells in the sdLN of 4× KO mice was significantly larger than that of 4× WT mice, indicating that the numbers of cells from 4× KO mice are more likely to expand. In contrast, a higher proportion of cells from 4× WT mice enter apoptosis and therefore are unable to respond to antigen. Together, these findings show that IL-10 in 4× WT mice acts to suppress CD4^+^ T cell proliferation and the ability of these cells to become activated and increases the likelihood of them entering apoptosis, ultimately leading to cell death.

The mechanism by which IL-10 may promote apoptosis and AICD is unclear. *In vivo*, IL-10 can regulate FasL expression, leading to increased T cell death (61), although others consider IL-10 to have a minimal role in driving CD4^+^ T cell AICD (25). In our studies of multiple schistosome infections, there was a significant decrease in the expression level of Fas mRNA in the sdLN of 4× KO mice, and there was significantly less apoptosis and cell death in 4× KO mice in the absence of IL-10, accompanied by an increase in the number of viable cells. Evidence for a change in FasL expression in KO mice is less convincing; consequently, another FasL-independent mechanism (25, 62, 63) involving IL-10 may contribute to apoptosis/cell death in our model.

In conclusion, we provide evidence for the induction of IL-10-associated apoptosis of CD4^+^ cells, which leads to the development of hyporesponsiveness in the lymph nodes draining the site of infection shortly after exposure to repeated doses of infective schistosome parasites. This occurs prior to the downregulation of immune responses due to schistosome eggs being released from adult worms during chronic infection (2, 6, 20, 29) and demonstrates that immune downregulation also operates during the early stages of parasite development and particularly in response to repeated exposure to infectious cercariae. The data presented here have wider relevance to the study of immune responses to other infectious pathogens, as most studies examine immune processes after only a single infectious dose. Our study shows that it is important to consider the effect of repeated/multiple exposures to different pathogens, as this is likely to be more significant in light of exposure to infectious agents in the natural world. While it is probable that there are several overlapping, and possibly redundant, mechanisms that underpin the development of CD4^+^ cell hyporesponsiveness, it is clear that IL-10 can have a central role. In this context, IL-10 was reported to be related to the intensity of human schistosomiasis (13, 64), which may be the cumulative result of repeated exposure to infectious cercariae as well as eggs released by adult worms.

## Supplementary Material

Supplemental material
