# Supplementary material for: CD4+ T Cell Hyporesponsiveness after Repeated Exposure to Schistosoma mansoni Larvae Is Dependent upon Interleukin-10
Source: Infect Immun. 2015 Mar 17;83(4):1418–30. doi: 10.1128/IAI.02831-14 (PMC4363412; doi:10.1128/IAI.02831-14)
Supplement: Supplemental material [file supp_83_4_1418__index.html]

CD4+ T Cell Hyporesponsiveness after Repeated Exposure to Schistosoma mansoni Larvae Is Dependent upon Interleukin-10 — Supplemental material 

# CD4+ T Cell Hyporesponsiveness after Repeated Exposure to Schistosoma mansoni Larvae Is Dependent upon Interleukin-10

## Supplemental material

**Files in this Data Supplement:**

- Supplemental file 1 -

  Fig. S1. Cytokine production by sdLN cells from mice exposed to a high- or low-dose 1× infection, and the proportions of CD8+ and B220+ cells in the sdLN of 1× and 4× infected mice. Fig. S2. Expression of PD1, PDL-1, and PDL-2 on myeloid cells in the sdLN. Fig. S3. APCs in the sdLN of IL-10 KO mice do not restore WT CD4+ responsiveness.

  PDF, 211K
