# Supplementary material for: CD4+ T Cell Hyporesponsiveness after Repeated Exposure to Schistosoma mansoni Larvae Is Dependent upon Interleukin-10
Source: Infect Immun. 2015 Mar 17;83(4):1418–30. doi: 10.1128/IAI.02831-14 (PMC4363412; doi:10.1128/IAI.02831-14)
Supplement: Supplemental material [file IAI.02831-14_zii999091152so1.pdf]

Figure S1

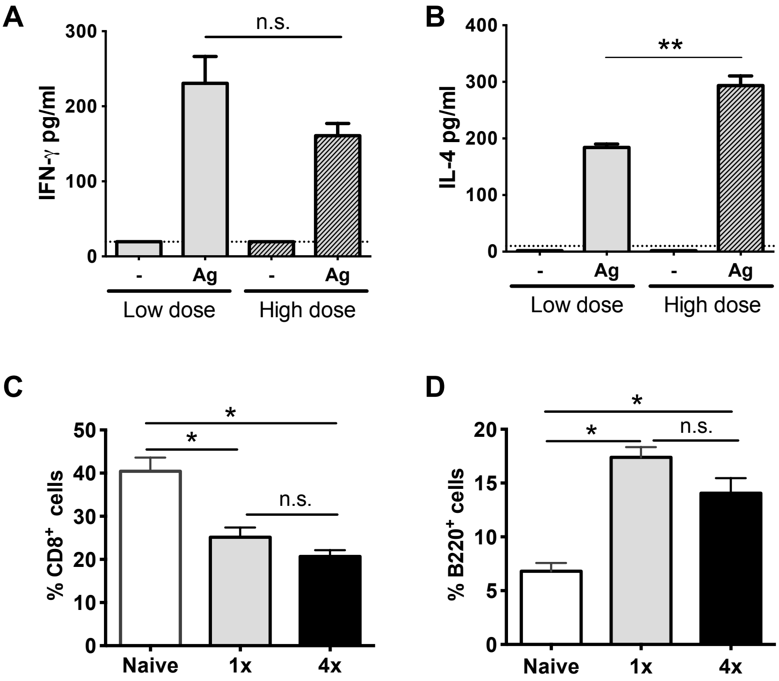

Cytokine production by sdLN cells from mice exposed to a high or low dose 1x infection, and the proportions of $CD8^+$ and $B220^+$ cells in the sdLN of 1x and 4x infected mice

Mice were exposed to a single low (150) or high (600) dose of cercariae. Cells from the sdLN were recovered four days after infection and single cell suspensions cultured *in vitro* with parasite antigen for 72 hrs. **A**. IFN-γ, and **B**. IL-4, in the culture supernatants detected using cytokine-specific ELISAs; values are means + SEM (n= 4-6 mice); dotted lines represents minimum level of cytokine detection. The proportion of **C**. $CD8^+$ and **D**. $B220^+$ sdLN cells from naive, 1x, and 4x mice obtained four days after the final infection. Statistical significance was tested using Mann Whitney U where * = $p<0.05$; ** = $p<0.01$; n.s. = $p>0.05$.

Figure S2

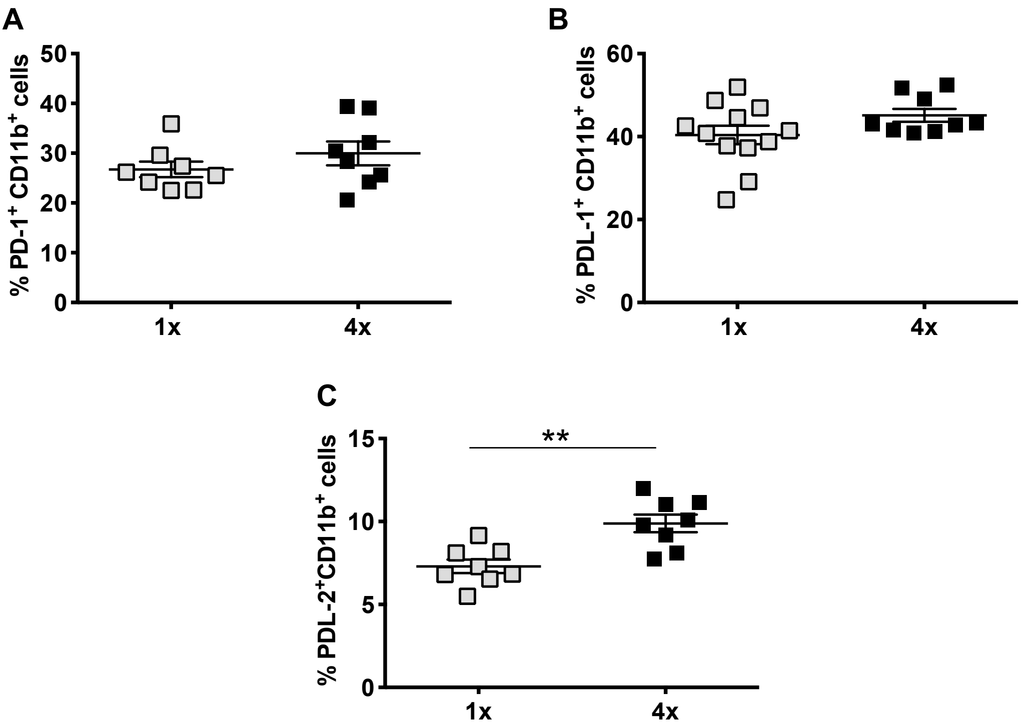

Expression of PD1, PDL-1, and PDL-2 on myeloid cells in the sdLN

Expression of **A**. PD1, **B.** PDL-1, and **C.** PDL-2 on $CD11b^+$ cells in the sdLN from 1x and 4x mice expressed as a proportion of total $CD11b^+$ cells, as measured by flow cytometry. Symbols are percent expression values for individual mice; horizontal bars represent the mean ± SEM (n=8 mice). Statistical significance was tested using Mann Whitney U where ** = $p<0.01$.

Figure S3

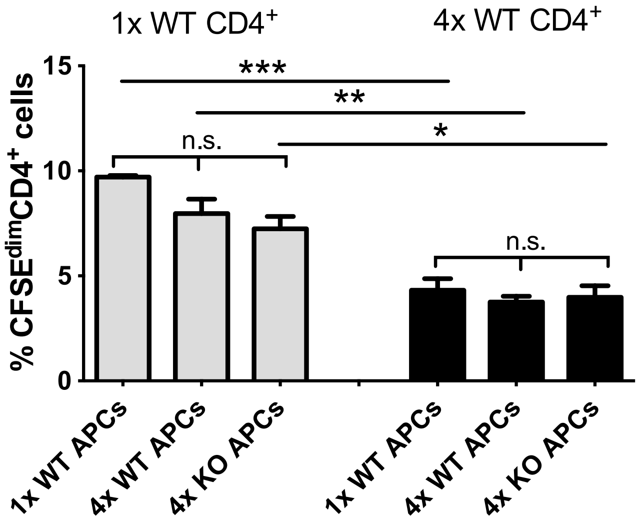

APCs in the sdLN of IL-10 KO mice do not restore WT CD4$^+$ responsiveness

CD4$^+$ cells from 1x and 4x infected WT mice were co-cultured with endogenous B220^{negative} APCs from the sdLN of 1x WT, 4x WT or 4x KO mice. Proliferation of CD4$^+$ cells cultured *in vitro* in the presence or absence of SSAP are shown as the proportion of CFSE^{dim} CD4$^+$ cells. Bars show the mean + SEM (n=4). Statistical significance of proliferation for 1x *versus* 4x CD4$^+$ cells was tested using Mann Whitney U where n.s. = $p>0.05$; * = $p<0.05$; ** = $p<0.01$; and *** = $p<0.001$
